# A structured multimodal teaching approach enhancing musculoskeletal physical examination skills among undergraduate medical students

**DOI:** 10.1080/10872981.2022.2114134

**Published:** 2022-08-22

**Authors:** Abdulaziz Z. Alomar

**Affiliations:** Division of Arthroscopy & Sports Medicine, Department of Orthopaedic Surgery, King Saud University, Riyadh, Saudi Arabia

**Keywords:** Multimodal teaching, musculoskeletal, peer-assisted learning, physical examination skills, undergraduate, video-based learning

## Abstract

Current evidence indicates that undergraduate medical students display deficits in musculoskeletal physical examination skills (MPES). While various instructional methods are recommended for teaching clinical skills, effective methods for teaching MPES have not been established. This study compared the effectiveness of a multimodal teaching approach incorporating video-based learning, interactive small-group teaching, hands-on practicing, peer-assisted learning, formative assessment, and constructive feedback with traditional bedside teaching in developing undergraduate orthopedic MPES. Participants were 151 fifth-year medical students divided into two groups. One group received multimodal teaching, and the other received traditional bedside teaching. In both groups, the participants learned how to physically examine the knee and shoulder. The primary outcome was objective structured clinical examination (OSCE) scores, while the secondary outcomes included teaching sessions’ total durations, facilitator’s demonstration time, participants’ practice time, and proportion of students with passing checklist scores and global ratings-based assessments for the two teaching approaches. The multimodal teaching group had significantly higher OSCE scores (checklist scores, global ratings, and passing rates; *p* = 0.02, 0.02, 0.01, respectively) than the comparison group. Individual OSCE component assessments showed significant improvements in the special musculoskeletal physical examination test. The overall duration and amount of participants’ hands-on time were significantly longer for the multimodal than for the traditional bedside teaching group *(p *= 0.01 and 0.01, respectively), and the facilitator’s demonstration time was significantly shorter (*p* = 0.01). The multimodal learner-centered teaching approach evaluated in this study was effective for teaching MPES. It appeared to maximize learner engagement through enhancing interactions and providing increased time to engage in hands-on practice. This teaching approach improved MPES levels, maximized teaching efficiency for scenarios with limited instruction time and resources, and enhanced competency of undergraduate medical students in performing special musculoskeletal physical examinations compared to traditional bedside teaching.

## Introduction

Undergraduate clinical training is critical for developing physical examination skills, which are essential for medical graduates to become primary healthcare providers. The development of adequate physical examination skills can allow primary healthcare providers to manage patients with musculoskeletal disorders who do not need specialized care, reducing the burden on hospitals [1,2]. Unfortunately, research on the development of musculoskeletal physical examination skills (MPES) among medical students is limited despite the curriculum-based implementation of clinical teaching [3–11]. Furthermore, undergraduate clinical teaching programs have focused primarily on general screening examination skills than specific physical examination skills [12] – an approach that could contribute to undergraduates’ lack of skills development. Additionally, musculoskeletal screening examinations may be insufficient for diagnosing and managing common orthopedic injuries and disorders without a specialized musculoskeletal examination [13].

Medical graduates must be competent in performing MPES, but these skills require a focused approach and include unique special physical examination tests that are not part of general screening musculoskeletal examinations [14]. In addition, these specialized tests require complex psychomotor skills that integrate multiple stimuli (i.e., tactile, visual, and auditory) as well as practical and theoretical knowledge that cannot be entirely learned through reading or observation [15].

Research suggests using lectures, bedside teaching, peer-assisted learning (PAL), case-based discussions, and simulation-based exercises to teach medical students how to conduct MPES [16–21]. However, the continuing deficits in medical students’ examination skills suggest that no single instruction method is ideal [16–19]. Moreover, several studies have provided novel education methods to help students gain the necessary skills, such as patient educators, multimedia computer-assisted learning, video-based learning (VBL), role-playing, and simulated patients (SPs) [22–25]. However, available data on the effectiveness of these methods of teaching MPES is limited, and any teaching methods for undergraduate medical students is likely to fail if it does not include hands-on practice [23].

Prior research has shown that improving students’ clinical skills requires the integrated and structured implementation of teaching methods that involve prior knowledge activation and briefing, bridging existing and new knowledge, elements of interaction and discussion, and opportunity for reflection and feedback [26–28]. Integrated and structured models can be effective means of teaching clinical skills if they maximize learner engagement, provide meaningful learning contexts, demonstrate the importance of imparting relevant knowledge before the teaching sessions (analogous to the flipped classroom) [29], deconstruct complex skills into small steps, and allow for interactive discussions, hands-on practice, performance assessment, and feedback mechanisms [16]. Yu et al. [30] deconstructed regional joint examinations into an introductory session covering the relevant musculoskeletal anatomy, joint range of motion, and palpation of the basic structures in a region, followed by a second session involving the special tests in MPES for each region. The authors reported significantly improved self-confidence related to MPES in the experimental group. Additionally, the objective structured clinical examination (OSCE) scores significantly improved in musculoskeletal stations with medium to large effect sizes across the different stations [30].

A multisensory approach to teaching clinical skills has been suggested for developing optimal learning [31]. This teaching approach integrates different stimulus mechanisms, such as visual and auditory methods, to increase the recognition and retention of information [31]. Diaz et al. [32] observed that teaching anatomy using extracurricular body painting was successful in engaging, motivating, and inspiring participants and first-year anatomy students to learn surface anatomy and develop their physical examination skills. Recently, Modica et al. [27] described a structured teaching approach for MPES to undergraduate medical students using a web-based musculoskeletal audio-visual tutorial, pathophysiology-focused cases, and facilitator preparation for conducting peer practice sessions. Although no significant differences in the OSCE-based assessment scores were observed, satisfaction and the additional benefit of a persistent resource were perceived by students learning through this teaching method [27].

To address undergraduate medical students’ need to learn MPES, we devised a structured multimodal teaching approach incorporating the benefits of the aforementioned methods. In this approach, students engaged in pre-session instructional VBL and handouts (i.e., flipped classroom), interactive small-group teaching, in-session live demonstrations, hands-on practice, PAL followed by formative assessment, and constructive feedback sessions. These methods were utilized sequentially.

Flipped classrooms, including VBL, can address time constraints since essential knowledge is imparted to the students before the physical session [29,33] and allow medical students to learn at their own pace and utilize class time to work through complex concepts [29]. PAL can effectively address the challenges of SPs for many students by practicing physical examination on peers [34]. Small group discussions provide an individualized approach to active learning compared to large group sessions [35]. A hands-on approach is appropriate for students practicing MPES to ensure they master the required skills [19]. In addition, a constructive feedback mechanism to check the effectiveness of the teaching session is required. Such a mechanism helps strengthen students’ skills and simultaneously address their weaknesses [36]. A formative assessment is a reliable method to assess the students’ skills and provide feedback after concluding a teaching session [24,37].

Students often experience challenges mastering MPES due to the lack of consistent and standardized teaching methods [38]. In addition, the complexity of MPES may not be addressed using a single instructional method. The total time given for classroom instructions is often limited in medical curriculum; thus, the approaches that keep students prepared prior to actual classroom instruction (i.e., flipped classrooms) can save time [29]. Further, student preferences for learning MPES vary considerably with most students preferring hands-on practice on real or simulated patients or peers, followed by multimedia and audio-visual tools [23]. Using multiple teaching modalities can potentially address the weaknesses in each method, which can provide flexibility for different learning preferences and a consistent and standardized plan for teaching MPES. Using a structured multimodal teaching approach as proposed above is one such step in improving MPES. This study aimed to evaluate the effectiveness of the proposed multimodal approach compared with traditional bedside approaches for teaching MPES, based on improvements in MPES competencies, OSCE assessment scores, and the proportion of competent students passing final clinical exams.

## Methods

### Study design and setting

After receiving approval from the institutional ethical committee (Approval No. 20/0002/IRB), this prospective comparative study was conducted at a medical school in Saudi Arabia, where the undergraduate medical curriculum is taught over seven years and includes a four-week orthopedics course in the fifth year. During those four weeks, the students exclusively receive instruction on orthopedics topics across the knowledge, skills, and attitudes domains. For the orthopedics course, the participants were divided into four groups, including 36–38 students each. These groups were divided into three smaller subgroups of 11–13 participants.

The needs assessment was performed by the medical education and orthopedic departments of the authors’ institution through surveys based on students’ perceptions of different methods of teaching MPES with additional feedback from the orthopedic faculties. A pilot study with 30 participants was conducted to ensure the study’s feasibility, appropriate interpretation, and administrative support .

### Participants

**Figure 1.**
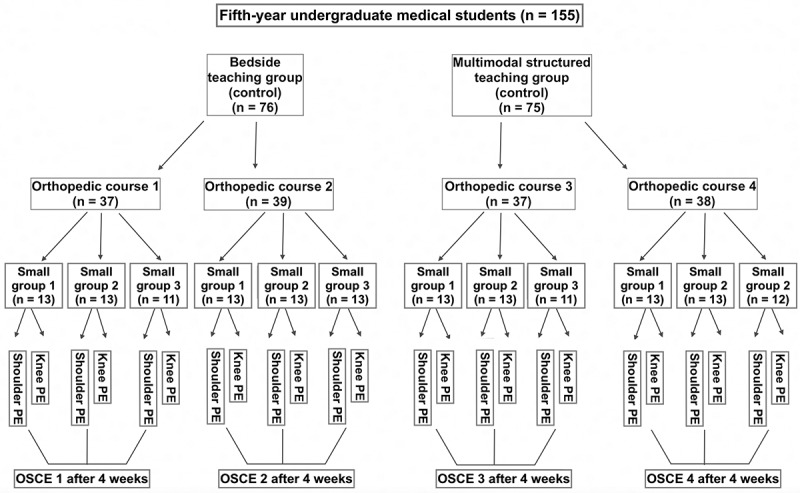
Flow of participants among the groups receiving multimodal and traditional teaching approaches. (OSCE: objective structured clinical examination, PE: physical examination).

Fifth-year male undergraduate students (*n* = 155) were recruited from the 2016–2017 academic cohort (Figure 1). Only one gender was examined since the studied institute has separate groups for male and female students in clinical rotation. The study was conducted during the male students’ rotation. Participants who missed the teaching sessions or did not agree to participate were excluded from the study.

### Intervention

The MPES teaching was conducted separately for each smaller subgroup. The standard framework of each MPES session consisted of a general examination (i.e., alignment/gait/inspection/palpation), range of motion assessment, special tests of individual joints with additional focus on communication skills, interpreting physical findings, and the ability to reach the diagnosis.

The duration allotted to physical examination instruction was the same for both groups (i.e., 2 hours) with the only differences being the teaching methodology of the two joint MPES sessions. One clinical session was conducted per day for every subgroup with different subgroups undergoing different joint examination sessions at a time. For the purpose of this study and to maintain uniformity, the same facilitator – an orthopedic consultant who specialized in shoulder and knee surgery – taught all the subgroups the correct knee and shoulder physical examination. Six small-group sessions were conducted for each knee and shoulder examination (Figure 1).

### Control group

In the traditional bedside teaching group, the facilitator provided a bedside demonstration of MPES using the standard framework of general examination (alignment/gait/inspection/palpation), range of motion assessment, and special tests with an SP, followed by interactive discussions involving communication skills and diagnosis formulation and to resolve the participants’ queries. Finally, the participants examined the SP.

### Intervention group

**Figure 2.**
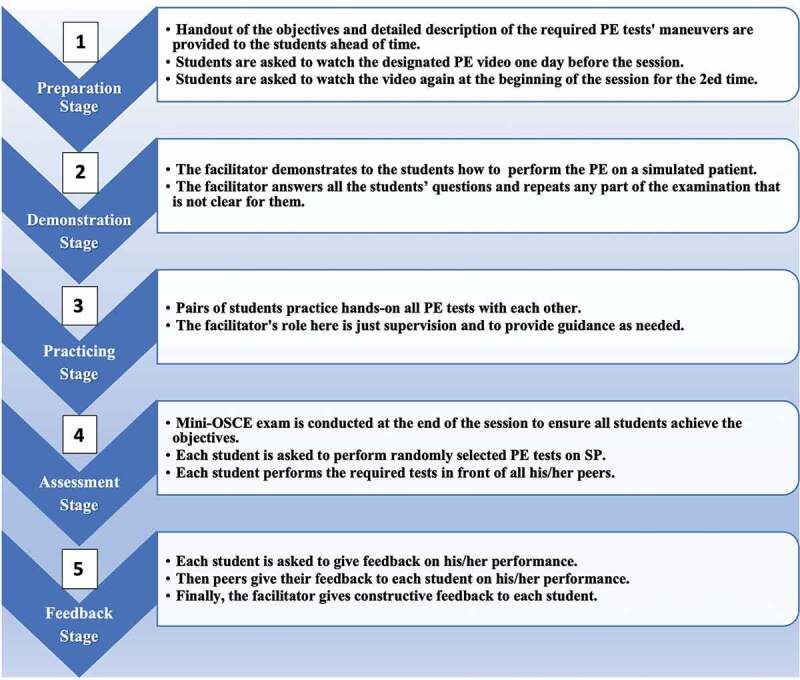
The structured multimodal approach for teaching musculoskeletal physical examination skills. (PE: physical examination, SP: simulated patient).

The structured multimodal teaching method sequentially combined several learning and assessment methods (Figure 2), which involved five stages, each of which involved one or more teaching modalities:

1. The Preparatory Stage: This phase used the flipped classroom method and stimulated MPES understanding by reading the study materials and the instructional videos that combined auditory and visual stimuli to improve MPES understanding prior to the physical teaching session. The students received a handout detailing the objectives and the required competencies (i.e., the ability to perform individual components of MPES correctly by the end of the session). It also described the required physical examination tests step-by-step according to the previously described standard framework. The facilitator created two separate instructional online YouTube videos of shoulder and knee examinations lasting 8 and 11 minutes, respectively. These videos covered the steps of general examination, range of motion, and special tests. Three expert orthopedic surgeons validated the video content (Supplementary Digital Files 1 and 2). Students were asked to watch the designated video one day prior to the teaching session and then again at the beginning of the session.
2. The Demonstration Stage: This stage combined bedside teaching with interactive small-group discussions. The facilitator demonstrated the physical examination step-by-step as described in the video using an SP and answered students’ questions. In addition, the facilitator stressed appropriate patient communication during the examination and diagnosis formulation based upon examination findings.
3. The Practice Stage: This stage focused on the students’ psychomotor domain while learning to perform MPES individually. The students were paired (PAL) to practice all physical examination steps on each other under the facilitator’s supervision.
4. The Formative Assessment: This step clarified MPES instructions among the students and ensured every student mastered the MPES special tests. The students were asked to perform special tests on the SP or their peer who acted as an SP. Each student in the subgroup was called by name and asked to perform special tests in front of their peers. If any student failed to perform the tests properly, then another student was called to perform the tests. The failing student was then asked to repeat the steps until they were performed correctly.
5. Constructive Feedback: In this last stage, each student, facilitator, and peer gave immediate constructive feedback to the other students on their performance. The feedback was based on Pendleton’s model, which involved a structured approach to improve students’ learning [36]. Pendleton’s model is described in four steps. First, the facilitator asks the student what went well and then tells the student what went well based on the facilitator’s observations. Furthermore, the facilitator asks the student what could be improved and then tells the student what could be improved based on the facilitator’s observations [36].

### Outcomes

The total teaching time per session, the facilitator’s demonstration time, and the participants’ clinical examination practice time were measured for each group. At the end of the four-week clinical orthopedics course, an OSCE was conducted for each group as part of a summative assessment (main outcome predictor). Each OSCE had two stations comprising a physical examination of the knee and shoulder with time slots of up to six minutes per station. Each participant performed knee and shoulder physical examinations at two stations, and each station had one clinical scenario and a well-trained SP with positive clinical findings.

One assessor per station and a total of eight different blinded assessors evaluated the participants performing the OSCE steps. The assessors were members of the teaching faculty in the orthopedic department (three assistant professors, three associate professors, and two professors selected for OSCE only). The facilitator who conducted the study was not one of the assessors; the assessors were not part of this study.

The OSCE assessment scores were presented in two ways: pre-validated checklist scores and global rating scales. Each station was assessed using a 10-point pre-validated checklist (Supplementary Tables 1 and 2) with individual scores (minimum = 0, maximum = 1) for each component of the physical examination and overall scores ranging from 0 to 10 for 10 components of each physical examination station (Supplementary Tables 1 and 2). The pre-validated checklist graded the participants’ performance at each OSCE component as 0 for ‘not performed/incorrectly performed,’ 0.5 for ‘partially correct/partially performed,’ and 1 for ‘correctly performed.’ The assessors provided global ratings that served as an overall assessment of the OSCE skills, graded from 1 to 5 (1 = *fail*, 2 = *borderline fail*, 3 = *borderline pass*, 4 = *clearly pass*, and 5 = *excellent*). The modified borderline standard-setting approach was used to label the participants as having ‘passed’ or ‘failed.’ The overall scores and pass-fail outcomes were compared between the two teaching methods. Additionally, the pass-fail results of the modified borderline approach based on OSCE scores were compared with those based on norm-referenced standard-setting scores, which the medical school routinely used; for the latter, the cutoff percentage score was fixed at 60%. The modified borderline group method was used; it is a criterion-based standard-setting method that is reliable for OSCE scoring of a large group. The available evidence supports the use of criterion-based standard-setting that includes all borderline grouping methods for OSCE assessment [39–41]. These methods are more objective, reproducible, transparent, and have good interobserver reliability. The findings support the use of borderline grouping methods considering their effectiveness in the skills assessment of the undergraduates [39–41].

### Statistical analysis

IBM SPSS Statistics for Windows, Version 23.0 (IBM Corp., Armonk, New York, USA) was used for the data analysis. The total teaching time per session, time the facilitator spent demonstrating the clinical examination, time the participants spent practicing the clinical examination, and the checklist- and global-rating-based OSCE scores for both groups were expressed as means (standard deviations [SD]). The total internal consistency of the OSCE scores was measured using Cronbach’s alpha. Since scores were not normally distributed according to the Kolmogorov-Smirnov test, Mann–Whitney U-test was used for independent samples to compare the quantifiable parameters between the teaching groups. An inter-rater reliability analysis using the Kappa statistic was performed to determine consistency among the eight raters. The interrater reliability for the raters showed very good agreement (κ = 0.86, *p* = 0.10, 95% CI [0.81–0.89]). The proportions of passing participants in each group were compared based on the checklist scores, and those with ‘clearly pass’ and ‘excellent’ global ratings were compared between groups using the chi-square test. Finally, the mean scores at the individual OSCE stations of the knee and shoulder physical examinations using the Mann–Whitney *U*-test were compared. Statistical significance was set at *p < .05.*

### Ethical approval and consent to participate

This study was approved by the King Saud University institutional review board (Approval Number: 20/0002/IRB). In addition, verbal and written informed consent was obtained from all participants.

## Results

A total of 155 students were enrolled in this study. Three students (one in the bedside teaching group, two in the multimodal teaching group) were excluded because of their absence from the teaching sessions, and one student in the multimodal teaching group did not agree to participate in the study. Among the remaining 151 students, 76 were assigned to the traditional bedside teaching group and 75 to the multimodal teaching group. The OSCE checklist items showed high internal consistency (overall Cronbach’s alpha = 0.89).

### Teaching time analysis

The overall teaching time per session was significantly higher in the multimodal group than in the traditional group. However, the time spent by the facilitator demonstrating the examinations was significantly higher in the traditional group. In contrast, the time the participants spent practicing clinical examinations was significantly greater in the multimodal group (Table 1).

**Table 1.** Comparison of time division among the two teaching methods-based groups.

Parameter	Traditional Model (*n* = 76) Mean (SD)	Multimodal Model (*n* = 75) Mean (SD)	*p*-value
Total time (min) needed for the session	60.1 (6.4)	85.9 (11.6)	.**01**
Time taken by the facilitator (min)	45.3 (12.5)	10.5 (2.2)	.**01**
Total time taken for hands-on practice (min)	12.1 (4.2)	59.5 (3.6)	.**01**

### Assessment scores

The overall checklist-based and global rating scores were significantly higher in the multimodal group than in the traditional group. A subgroup analysis of the individual checklist scores on the general physical examination portion (inspection, palpation, and range of movement), special tests, communication skills, and the ability to reach diagnosis revealed significant differences between the groups in the combined assessment of the special tests. These differences remained significant for separate assessments of the knee and shoulder physical examinations (Table 2).

**Table 2.** Comparison of OSCE results between the two study groups.

OSCE results	Assessment Type	Traditional Model (*n* = 76) Mean (SD)	Multimodal Model (*n* = 75) Mean (SD)	*p*-value
Combined shoulder and knee assessment	Checklist Scores	6.90 (0.87)	7.99 (0.91)	**0.02**
	Global Ratings	2.77 (0.71)	3.70 (0.82)	**0.02**
Knee assessment	Checklist Scores	6.80 (0.62)	8.03 (0.83)	**0.01**
	Global Ratings	2.66 (0.21)	3.71 (0.19)	**0.01**
Shoulder assessment	Checklist Scores	6.91 (0.52)	7.91 (0.74)	**0.01**
	Global Ratings	2.88 (0.15)	3.69 (0.18)	**0.01**

**Figure 3.**
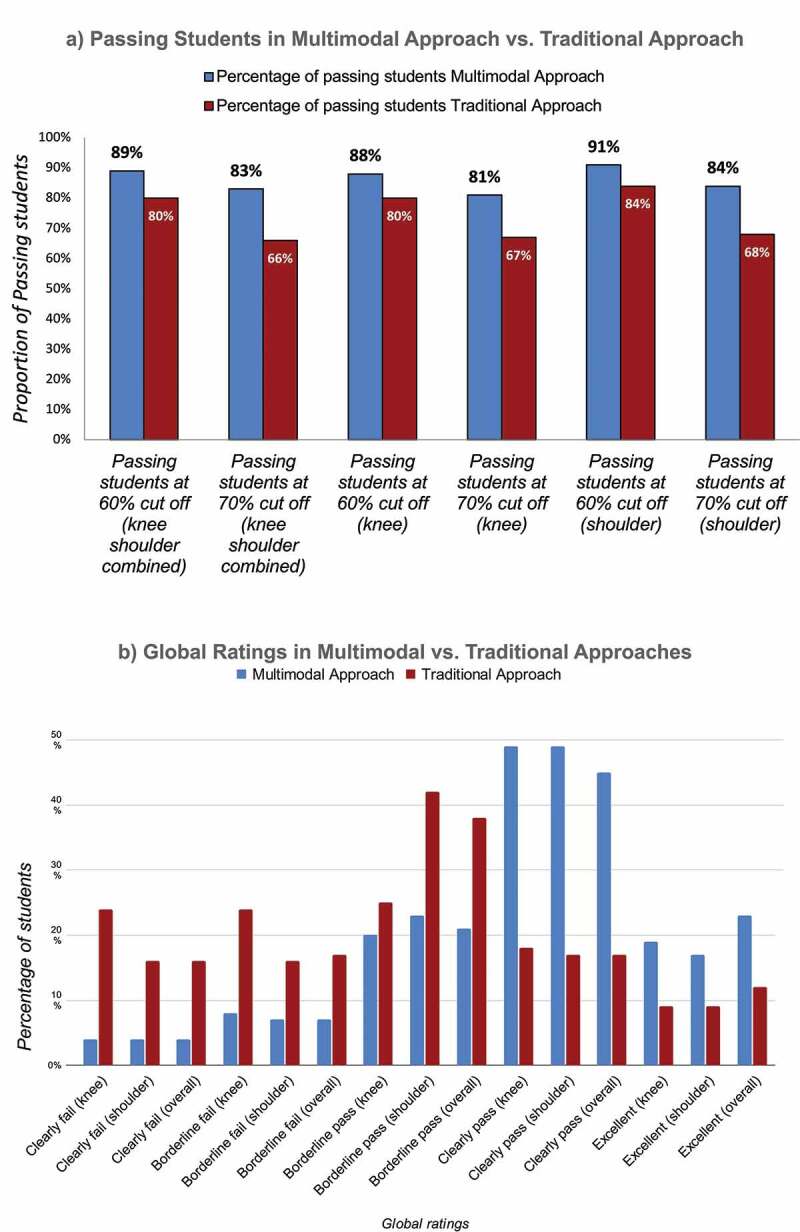
(a) Proportion of passing students in the multimodal and traditional teaching groups at the institutional cutoff score of 60% and modified borderline grouping-based cutoff score of 70% for knee and shoulder OSCEs, combined and separately. (b) Global-ratings-based assessment of students among the multimodal and traditional bedside teaching approaches.

In the norm-referenced approach (cutoff rate 60%), no significant differences were observed in the proportion of overall pass rate of participants in the combined and separate assessments of knee and shoulder physical examinations. However, in the modified borderline approach (cutoff rate 70%), there was a significantly higher proportion of overall passing of participants in the multimodal group than in the traditional group, and significant differences were observed in both the combined and separate assessments of knee and shoulder physical examinations. Regarding global-ratings-based assessments, a significantly higher proportion of students had ‘clearly pass’ or ‘excellent’ global ratings in the multimodal group than in the traditional group in both the combined and separate assessments of knee and shoulder OSCEs. Most students had ‘borderline pass’ global ratings in the traditional bedside teaching group for knee and shoulder assessment separately as well as overall. Conversely, most students had ‘clearly passed’ global ratings in the multimodal teaching group for knee and shoulder assessment separately and overall. Figure 3 and Table 3 present these results.

**Table 3.** Significance of proportion-based differences between structured multimodal and traditional bedside teaching groups.

Proportional Parameters		Outcome	*p*-value
‘Clearly pass’ or ‘excellent’ global ratings	Overall	Higher proportion in the Multimodal method	.**01**
	Knee	Higher proportion in the Multimodal method	.**01**
	Shoulder	Higher proportion in the Multimodal method	.**01**
Passing students at 60% cutoff of checklist scores	Overall	Higher proportion in the Multimodal method	.12
	Knee	Higher proportion in the Multimodal method	.19
	Shoulder	Higher proportion in the Multimodal method	.23
Passing students at 70% cutoff of checklist scores	Overall	Higher proportion in the Multimodal method	.**02**
	Knee	Higher proportion in the Multimodal method	.**04**
	Shoulder	Higher proportion in the Multimodal method	.**04**

### Analysis of OSCE scores for individual joints

Compared with the traditional group, the multimodal group showed significantly higher checklist-based scores for individual OSCE questions in five of six special tests of the knee joint examination (Supplementary Table S1) and four of five special tests for the shoulder examination (Supplementary Table S2). However, no statistically significant differences were found between the groups regarding the general examination, range of motion, communication skills, and diagnosis formulation for both joints.

## Discussion

The current findings highlight the advantages of a multimodal teaching method for teaching and learning MPES. Comparisons of the two teaching models showed a notable change in special musculoskeletal physical examination test skills.

The current study findings suggest a shifting paradigm in teaching MPES that moves from traditional bedside teaching to a multimodal approach, similar to how medical education evolved from the traditional Flexnerian model to the competency-based curriculum [42]. Traditional teaching models in medical education include more observation and less participation [43,44]. Additionally, the domains related to communication, doctor-patient relationship, ethics, and professionalism have not been addressed effectively in traditional curricula [45]. MPES teaching methods are likely similar, considering the ample evidence demonstrating that important special tests in musculoskeletal examination are often not performed and remain undocumented by doctors [46–48]. Teaching-learning methods in competency-based curricula should be learner-centered [42]. Furthermore, to make learning more effective, such methods should use multiple modalities to address all three learning domains (i.e., knowledge, skills, and attitude) [49,50]. Fleming et al. [51] suggested that students learn by visual, auditory, reading/writing, and kinesthetic sensory modalities. The fact that most students benefit from more than one learning style has been well established, suggesting the need for a multimodal teaching approach [52,53]. Previous researchers have advocated using multimodal methods to teach clinical competencies [52–54].

In this study, the performance on the general part of the physical examination was satisfactory for both groups, which may be explained by the fact that the psychomotor skills needed for this part were relatively simple. However, there were significant differences between the two groups regarding special test performance because they assess complex psychomotor skills covering all three learning domains. Therefore, the use of multiple modalities can help achieve MPES-related competencies. However, the evidence for a multimodal approach for MPES teaching is limited. There are several methods for medical teaching; however, active learning methods are preferable for competency-based teaching that includes some student participation components [42]. However, combining different learning methods may strengthen students’ learning and address differences in their learning styles [52,55,56]. While the facilitators can include multiple teaching methods for different competencies, those involving the MPES should be tailored to the methods that allow students to perform the desired skills correctly. Practicing on peers/instructors/SPs/real patients has been identified as the preferred learning modality for musculoskeletal learning among medical students; thus, such methods should be incorporated into MPES instruction [23]. Our findings suggest that the proficiency of MPES performance may not be sufficiently addressed using traditional bedside teaching alone. In a traditional curriculum, simply demonstrating the MPES by a teacher with or without individual student participation cannot ensure that all students will perform the MPES in the demonstrated manner and have the chance to perform all required skills correctly. Therefore, traditional bedside teaching is more teacher-centered than learner-centered, as the learner may acquire the knowledge of the examination but may not be able to perform the clinical examination skills as taught. The multimodal approach described in this study is learner-centered and provides a standardized framework for teaching and learning MPES to ensure that all students can perform predefined MPES competencies successfully and efficiently. The different teaching modalities incorporated in the different phases of the multimodal approach were carefully planned to ensure that all students could achieve the desired outcome. Since traditional bedside teaching is a widely used method for MPES teaching worldwide, there is a need to shift to a multimodal structured approach that can potentially help improve MPES performance among medical students.

Our study also suggests that the conventionally used norm reference-based standard settings may not be suitable for MPES assessment. While no significant differences were found between the two teaching groups and the norm reference-based standard (i.e., institutional cut-off based), significant differences were observed with modified borderline-based standard setting. The cut-offs could explained the differences in the two standard setting methods (~60% norm reference, ~70% modified borderline method). The modified borderline approach is a criterion-referenced standard setting based on the minimally acceptable competency level to pass each step in the exam. Comparing the individual components of the checklist-based score, the special test scores were significantly lower in the bedside teaching group than in the multimodal group. Therefore, candidates should be divided into the borderline pass and fail groups by experts’ global ratings to better assess MPES [57]. We used the modified borderline group method that is reliable for the OSCE scoring of a large group, and the mean borderline scores are used to calculate the cut-off scores [39]. A criterion-referenced standard setting compares the performance against a set standard or threshold and is preferable for competency-based learning since the outcomes should be measurable. However, norm-based assessments compare students’ performance with that of their peer groups. Such an assessment may not reflect what a student can or cannot do and cannot determine whether the desired competency outcomes were met. Our study supports the use of criterion-based assessment for MPES assessment since a norm-based standard setting is not accurate when assessing a competency. Concerning this study’s findings, we feel that students’ better special test performance in the multimodal teaching group was because each student had the opportunity to perform all required tests with supervision, unlike the traditional bedside teaching group where the students had voluntary participation only.

Few previous studies have attempted to investigate the role of multimodal teaching in physical examination skills among medical students. Allen et al. [58] analyzed OSCE scores after structured history and clinical examination exercises for back pain evaluation and suggested the lack of new modalities to stimulate audiovisual learning and hands-on practice and feedback mechanisms, which resulted in the suboptimal OSCE performance of medical students. Hands-on practice added to structured teaching, especially with supervision, has been found to be effective for various skills [59]. Modica et al. [27] used a similar approach for musculoskeletal diagnosis- and pathology-focused cases; however, no OSCE score improvements were observed. While there was a component of potentially biased assessment in OSCE scores, the students were satisfied with the structured teaching approach.

Although longer teaching sessions were observed in the multimodal group than in the traditional group, multimodal facilitators spent less than one-fourth of the time demonstrating skills as in the traditional group. Most additional time for the multimodal group was devoted to participants’ hands-on practice, indicating better opportunities for students’ MPES learning. Thus, bedside teaching may be a teacher-centered approach, while multimodal teaching is learner-centered. Traditional methods are predominantly didactic [44]; the learner-centered approach focuses on methods to provide better learning opportunities to students with their active participation [44].

The flipped classroom in the preparatory phase (i.e., VBL and instructional handouts) imparted basic knowledge to participants and outlined the expectations for them during the examinations [29]. This prior knowledge may have made the demonstration easier for the facilitator since students may have been able to adapt most of what they had learned from the VBL [26].

It has been stressed that students’ learning methods vary widely. A well-known VARK adult learning style consists of reading/writing, auditory, and visual learners [50]. The multimodal approach provides an integrated approach that caters to each student’s learning style with different components promoting visual, auditory, and text-based learning. Furthermore, the hand-on segment adds the element of learning by doing, which can potentially boost students’ confidence [19].

The current study using multiple sensory stimulations provides multiple opportunities for students to grasp MPES in their preferred ways of learning. Information from one modality can influence information processing in another modality. Information from different sensory modalities can be combined into a single multisensory event [60]. As far as human learning behavior is concerned, a single sensory teaching methodology may not facilitate optimal learning; multisensory stimulation is important for optimally developing skills and learning [31]. Some studies have suggested improving clinical skills through multisensory stimulation [60,61].

In contrast, students in the traditional group took approximately one-fifth of the time to conduct physical examinations as the multimodal group. One reason for this might be that the participants in the traditional group were less confident about performing the physical examination and feared confrontation with peers or making mistakes while being observed by peers. The multimodal method may help address these participation-related issues with its multifaceted approach.

Specifically, the research showed that providing participants with a preview of the lessons through VBL could strengthen their knowledge and reduce the time required to understand concepts. Strengthened knowledge develops participants’ interest in performing examinations and resolves their doubts [62]. Moreover, studies suggest that VBL can be an effective method for teaching physical examination and clinical skills to large student groups and is perceived well by the students [63,64]. It has proven to be a cost-effective and scalable resource for teaching clinical skills [28,65,66]. Unlike a lecture/demonstration, which progressively moves to new topics, students can view each step repeatedly until they are confident of doing it independently [66]. Easy access to instructional videos can help students retain knowledge, develop self-confidence, practice independently, and reduce errors [28,65]. However, despite its obvious advantages, VBL alone is unlikely to be enough for students to develop MPES skills; they also need hands-on practice. Accordingly, a blended method is proposed rather than a single method based solely on VBL.

Interactive small-group teaching is a learning method that uses a student-centered approach [67]. Learning in smaller groups enhances individual attention, student-teacher interactions, and hands-on experiences [35]. Small-group teaching has been shown to be advantageous for MPES teaching, leading to improved student satisfaction and scores [68,69]. In the present study, each small group had a maximum of 12 participants, which improved each participant’s chance of receiving appropriate attention and getting involved in group activities. For both groups, teaching sessions were conducted using only small-group teaching, which might explain why comparable scores in general physical examination skills were observed between the two groups. The teaching-related modifications introduced by the multimodal approach might also have contributed to the enhanced special test skills observed in the multimodal group.

In PAL, participants assisted each other in teaching and learning in a supportive manner. PAL addresses the limitations due to the unavailability of SP for all sessions as students practice clinical examination steps with their peers [34]. It adds the element of practice and allows each student to build confidence in conducting physical examinations in front of their peers. Several studies have confirmed the effectiveness of PAL in teaching clinical skills [70–72]. Burke et al. [73] and Graham et al. [74] suggested that PAL could effectively improve MPES in medical students and that students could act as effective trainers. The current study’s findings supported this; the scores for special test skills, which generally require more precise understanding and practice from students, were better for the multimodal group in which PAL was an integral component. PAL supports learners’ cognitive, psychomotor, and affective development [75]. Students taught with a multimodal approach might be more comfortable practicing and performing in front of their peers, which should translate to greater patient confidence.

To assess whether the study’s participants achieved each session’s main objectives, formative assessments were used at the end of each session because these were considered an appropriate tool for this study. These formative assessments also provided facilitators and participants with immediate objective feedback. Timely feedback can help teachers and students identify corrective actions to improve learning [76]. Pendleton’s feedback model used in this study ensured the bidirectional flow of information, which helps improve student learning outcomes [36]. Such a feedback process is in line with the concept of feedback literacy. Feedback literacy has a framework of four interrelated features: appreciating feedback, making judgments, managing effect, and taking action [77]. The combination of facilitators’ and students’ observations help in generating constructive feedback. As the feedback was immediate, the students had the advantage of resolving their doubts and correcting any deficits immediately. However, further evidence would be required to understand how the feedback strengthening impacts the students’ performance in MPES.

Unlike specialty residents or trainees, undergraduate medical students’ training and practice are not regular and are bound to change with every specialty-specific training period. The multimodal teaching approach improves students’ practice time and likely helps improve OSCE performance. However, such an approach may not have the same outcomes for specialty trainees, who learn skills through regular practice and have much longer practice exposure. Similarly, those fields that involve more hands-on practice, such as physiotherapy, may not equally benefit from a new approach considering that hands-on training is an integral part of the specialty. For example, in a randomized control group Hossain et al. [78] found no significant increase in knowledge or confidence among physiotherapy students regarding the physiotherapy management of spinal cord injuries by an integrated online module course, and they were not more satisfied with the learning experience.

### Limitations

This study had some limitations. First, the design was not randomized and had a planned sequence of teaching group allocations. However, this nonrandomized sequence did not entail preferential group allocation; instead, group allocation was decided by the administrative plan, and students were unaware of their group before participating. Second, the effectiveness of the structured multimodal MPES teaching method on knee and shoulder joint skills after only four weeks of implementation was evaluated – future research should conduct more extensive analyses of implementation feasibility in curricula and its long-term implications for student performance. Third, the OSCE was not conducted concomitantly in either group; the OSCE’s different timings and questions might have impacted the participants’ performance. Fourth, the study could not predict which step of the multimodal teaching approach was specifically responsible for improving students’ performance in OSCE. A combination and sequence of all the involved methods may have contributed to the outcomes.

Fifth, the study evaluated only male students’ skills, and a uniform gender distribution could have different results. The evidence regarding the gender difference in the acquisition of MPES has not been investigated previously. The available evidence regarding clinical examination skills among male and female students suggests differences in their approaches. These differences could be influenced by the gender profile of facilitators, role models, patients’ profiles, type of clinical examinations, and other factors yet to be established [79,80]. While the gender differences cannot be denied, involving students of one gender could potentially standardize these factors among the participants and project the effects of the teaching approach in general.

Lastly, the students in the multimodal teaching groups might have watched examination videos multiple times and practiced clinical examinations. This could potentially have resulted in higher assessment scores in the intervention group. However, increased video watching and practicing would ultimately strengthen the modified approach’s purpose and would benefit the students.

Despite these limitations, this research shows that a structured multimodal teaching approach could be more effective than the traditional bedside method in improving medical students’ substandard performance in MPES. Further studies are required to bolster and expand upon the current evidence.

## Conclusions

The described learner-centered, multimodal teaching approach can improve the competency of undergraduate medical students in MPES performance. This structured teaching approach can help improve teaching efficiency considering the limited instruction time and build interest among students since they spend more time practicing than understanding. Although bedside teaching-trained students may remain competent in general physical examination, diagnosis formulation, and communication skills, students trained with the structured approach perform better in musculoskeletal physical examination special tests. A curriculum-based implementation of such an approach can potentially improve MPES among undergraduate students.

## Lessons for practice

- The structured multimodal teaching method was more effective than the single method teaching approach in improving medical students’ MPES.
- Incorporating VBL, PAL, and hands-on practice in physical examination teaching could maximize learner engagement, provide meaningful learning contexts, improve MPES performance and students’ practice time, and maximize teaching efficiency.
- Formative assessments followed by constructive feedback are appropriate tools that can be used at the end of a teaching session to assess whether students achieved the required competencies and can help teachers and students identify corrective actions to improve learning.

## Supplementary Material

Supplemental Material
