# Supplementary material for: A structured multimodal teaching approach enhancing musculoskeletal physical examination skills among undergraduate medical students
Source: Med Educ Online. 2022 Aug 22;27(1):2114134. doi: 10.1080/10872981.2022.2114134 (PMC9466621; doi:10.1080/10872981.2022.2114134)
Supplement: Supplemental Material [file ZMEO_A_2114134_SM8324.zip › supplementary files/additional_file_1 (1).docx]

**Supplementary Table 1**

Knee joint OSCE examination components: Comparison of multimodal and traditional bedside teaching groups of 151 undergraduate medical students

| **OSCE components** | **Traditional model**  **(*n* = 76)**  **Mean (SD)** | **Multimodal method**  **(*n* = 75)**  **Mean (SD)** | ***p*-value** |
| --- | --- | --- | --- |
| General examination (alignment, gait, inspection, palpation) | 0.97 (0.14) | 0.97 (0.11) | .99 |
| Range of motion (active and passive) | 0.96 (0.18) | 0.97 (0.13) | .81 |
| Special test 1(knee effusion): milking test | 0.72 (0.22) | 0.89 (0.15) | **.02** |
| Special test 2 (menisci): joint line palpation | 0.65 (0.24) | 0.90 (0.13) | **.01** |
| Special test 3 (ACL): Lachman or ADT test | 0.65 (0.19) | 0.92 (0.18) | **.01** |
| Special test 4 (PCL): PDT | 0.58 (0.21) | 0.80 (0.20) | **.01** |
| Special test 5 (MCL): valgus stress test | 0.45 (0.27) | 0.61 (0.17) | **.02** |
| Special test 6 (LCL): varus stress test | 0.41 (0.26) | 0.47 (0.32) | .30 |
| Communication skills | 0.94 (0.22) | 0.95 (0.18) | .86 |
| Identify physical findings and reach differential diagnoses | 0.45 (0.49) | 0.47 (0.49) | .94 |
| Abbreviations: ACL: anterior cruciate ligament; ADT: anterior drawer test; LCL: lateral collateral ligament; MCL: medial collateral ligament; OSCE: objective structured clinical examination; PCL: posterior cruciate ligament; PDT: posterior drawer test; SD: standard deviation. | | | |
