# Supplementary material for: A structured multimodal teaching approach enhancing musculoskeletal physical examination skills among undergraduate medical students
Source: Med Educ Online. 2022 Aug 22;27(1):2114134. doi: 10.1080/10872981.2022.2114134 (PMC9466621; doi:10.1080/10872981.2022.2114134)
Supplement: Supplemental Material [file ZMEO_A_2114134_SM8324.zip › supplementary files/additional_file_2.docx]

**Supplementary Table 2**

Shoulder joint OSCE examination components: Comparison of multimodal and traditional bedside teaching groups of 151 undergraduate medical students

| **OSCE Components** | **Control group**  **(*n* = 76)**  **Mean (SD)** | **Multimodal method**  **(*n* = 75)**  **Mean (SD)** | ***p*-value** |
| --- | --- | --- | --- |
| Inspection | 0.99 (0.08) | 0.98 (0.10) | .64 |
| Palpation | 0.97 (0.11) | 0.99 (0.06) | .18 |
| Range of motion (active and passive) | 0.82 (0.27) | 0.87 (0.25) | .33 |
| Special test 1 (supraspinatus): Jobe’s test or empty can test | 0.67 (0.14) | 0.88 (0.09) | **.01** |
| Special test 2 (infraspinatus): resisted external rotation | 0.66 (0.19) | 0.90 (0.11) | **.01** |
| Special test 3 (subscapularis): lift-off or belly press test | 0.61 (0.12) | 0.81 (0.18) | **.01** |
| Special test 4 (recurrent instability): apprehension test | 0.55 (0.14) | 0.66 (0.10) | **.01** |
| Special test 5 (impingement syndrome): Neer sign or Hawkins test | 0.39 (0.24) | 0.49 (0.31) | .06 |
| Communication skills | 0.97 (0.15) | 0.98 (0.13) | .57 |
| Identify physical findings and reach differential diagnosis | 0.37 (0.26) | 0.47 (0.40) | .18 |
| Abbreviations: OSCE: objective structured clinical examination; SD: standard deviation. | | | |
